# Novel green solvent-in situ ultrasound synergistic extraction of phenolic acids from *Lonicera japonica* thunb

**DOI:** 10.1016/j.ultsonch.2026.107779

**Published:** 2026-02-13

**Authors:** Cheng Liu, Qian Zhao, Xin Ma, Shuxin He, Xiaochuan Zou, Fangyuan Gong, Ying Liu, Jie Lei, Zhengwei Xiong, Jiayun Liu

**Affiliations:** a Chongqing Field Scientific Observation and Research Station for Authentic Traditional Chinese Medicine in the Three Gorges Reservoir Area, Chongqing University of Education, Chongqing 400067, China; b College of Biological and Chemical Engineering, Chongqing University of Education, Chongqing 400067, China; c Department of Clinical Laboratory, Xijing Hospital, Fourth Military Medical University, Xi’an 710032, China

**Keywords:** TDES, In situ ultrasound-synergized extraction, *Lonicera japonica Thunb.*, Phenolic acids, RSM-ANN-GA

## Abstract

A novel ternary deep eutectic solvent combined with in situ ultrasound–synergized extraction (TDES–ISUSE) was designed and implemented for the efficient recovery of four phenolic acids from *Lonicera japonica* Thunb. From twenty synthesized TDES formulations, the ternary system comprising choline dihydrogen citrate, lactic acid, and urea (Chd:Lac:Ure) demonstrated superior extraction performance. Process optimization via an integrated RSM–ANN–GA approach established the following optimal conditions: water content 37%, liquid/solid ratio 28 mL/g, ultrasound time 30 min, vortex time 12 min, and ultrasound power 240 W. The total yield of the four phenolic acids reached 90.66 ± 1.46 mg/g, significantly surpassing conventional extraction techniques. Chemical profiling by UHPLC–Q–TOF–MS in both positive and negative ionization modes led to the identification of 19 compounds, categorized as seven phenolic acids, three flavonoids, one iridoid, one terpenoid, five saponins, and two fatty acids, while a dedicated HPLC method was validated for quantitation of the four target phenolic acids. The characteristic FT–IR signatures corroborated the successful formation of the TDES. The enhanced cell–wall disruption was directly visualized by SEM, while molecular dynamics simulations elucidated the improved solvation of phenolic acids within the TDES system at the molecular level. Antioxidant assays further indicated notable radical–scavenging activity of the extract. Collectively, this study presents an efficient, environmentally benign, and reproducible strategy for the extraction of bioactive phenolic acids from *L. japonica*.

## 1 Introduction

*Lonicera japonica* Thunb., a woody vine belonging to the Caprifoliaceae family, is predominantly distributed in the South-Central and Southwest regions of China [1]. With a medicinal history spanning over 1500 years, it is widely recognized as a classic traditional Chinese medicine (TCM). Its therapeutic applications have been extensively documented in ancient TCM classics and modern pharmacopoeias, particularly for the treatment of heat–toxin–induced dysentery, wind–heat common cold, and other inflammation–related disorders [2]. Beyond its clinical use, *L. japonica* is also utilized as a traditional edible resource in Chinese dietary culture [3]. The dried flower buds are commonly brewed into herbal tea to alleviate summer heat, and are further incorporated into pastries, soups, and functional foods owing to their agreeable flavor and potential health–enhancing properties [4].

Phytochemical investigations have established that *L. japonica* is rich in phenolic acids, flavonoids, volatile oils, and other bioactive compounds. Among these, phenolic acids are considered the principal bioactive constituents, characterized by their favorable solubility in conventional extraction solvents [5]. Such solvents can be efficiently permeated and agitated under ultrasonic cavitation, which promotes the release of intracellular phenolic acids through mechanical disruption of the cell wall [6], [7]. This process enhances both the extraction yield and the selectivity for target phenolic acids. Notably, phenolic acids exhibit significant pharmacological activities, including potent antioxidant, antibacterial, and antiviral effects [8], [9]. With advancing research in natural medicine, *L. japonica* has attracted increasing attention from the scientific community in recent years.

Commonly used solvents for extracting bioactive compounds from natural products include methanol, ethanol, and acetonitrile. However, organic solvents suffer from notable limitations, such as ecosystem contamination, excessive consumption, and potential health risks [10]. Therefore, researchers are striving to develop novel green solvents for sustainable bioactive compound extraction. Deep eutectic solvents (DESs), a new class of solvents formed by mixing hydrogen bond acceptors (HBAs) and hydrogen bond donors (HBDs) at an appropriate molar ratio, offer a range of promising advantages [11], [12]. They feature cost-effectiveness, facile fabrication, eco-friendliness, biosafety and renewability [13]. Compared with conventional solvent systems, DESs demonstrate higher efficiency, lower cost, and a more favorable environmental profile [14], [15]. The classic formulation of a DES involves a binary mixture of a single HBA and a single HBD. A ternary deep eutectic solvent (TDES), by contrast, integrates a third component, where all three moieties interact through intermolecular hydrogen bonding [16]. This additional component strengthens the overall hydrogen-bonding framework, resulting in enhanced solvent stability [17]. For instance, Ran et al. [18] reported the use of a ternary DES composed of glycerol, malic acid, and malonic acid for extracting naringin from *Exocarpium Citri Grandis*, achieving an optimal yield of 175.30 ± 2.94 mg/g—approximately 30% higher than conventional solvents. Wang et al. [19] developed a TDES composed of betaine, ascorbic acid, and glycerol for the efficient extraction of squalene from *Strobilanthes tonkinensis*. Under optimized conditions, the method achieved a yield of 331.79 μg/g, representing a 2.9% enhancement over conventional n–hexane extraction. A Capitalizing on the proven efficacy of deep eutectic solvents for bioactive extraction, the present work was designed to formulate a novel TDES. The system, composed of choline dihydrogen citrate, lactic acid, and urea in a 1:2:2 M ratio, exhibits the perfect efficiency.

Ultrasonication, which enhances mass transfer via cavitation–driven shear forces, serves as a key enabler of green extraction. Here, it is integrated with in–situ extraction in a novel dual–mode strategy to advance sustainable processing. This strategy provides a combination of speed, excellent reproducibility, operational simplicity, and heightened efficiency [20]. The extraction mechanism is fundamentally governed by cavitation, wherein ultrasonic pressure fluctuations induce periodic rarefaction-compression cycles in the liquid phase. Through the pressure oscillations, microbubbles are nucleated and undergo violent collapse in the liquid medium [21]. Consequently, this synergistic mechanism thereby allows for the effective liberation of phytochemicals from the plant matrix.

Conventional response surface methodology (RSM) often falls short in capturing the intricate, nonlinear interdependencies and higher-order interactions among extraction parameters, leading to constrained predictive performance [22]. To address this limitation, we developed an integrated computational framework that synergistically combines RSM, artificial neural networks (ANN), and genetic algorithms (GA). The hybrid approach leverages RSM for efficient experimental design and preliminary modeling, employs ANN to accurately map complex nonlinear relationships, and utilizes GA for global optimization of the parameter space, thereby substantially enhancing both prediction accuracy and process optimization in multifaceted extraction systems.

This work developed a novel TDES and a innovative extraction strategy to extract phenolic acids from *L. japonica*. First, twenty candidate TDESs were prepared and then systematically assessed to screen for the most performant formulation. Subsequently, an integrated RSM-ANN-GA framework was developed to confirm the optimal extraction conditions, and the performance of the proposed method was evaluated against conventional extraction techniques. Additionally, UHPLC–Q–TOF–MS was applied for the first time to comprehensively characterize the bioactive constituents in *L. japonica*. Furthermore, the newly formulated TDES was characterized by FT–IR, while SEM and molecular dynamics (MD) simulations were utilized to elucidate extraction mechanism at microscopic levels. Finally, the antioxidant activity of the obtained extracts was systematically assessed. In summary, this study introduces an innovative solvent and extraction methodology, providing a valuable reference for the further development and utilization of *L. japonica* in pharmaceutical and food applications.

## 2 Materials and methods

### 2.1 Raw materials

*L. japonica* was collected from Hongta Town, Fang County, Shiyan City, Hubei Province in June 2025 and was botanically authenticated by Researcher Liu Yanqin from the Chongqing Institute of Medicinal Plant Cultivation. The dried flowers were ground and sieved through a 40–mesh screen. All chemical reagents used in this study were purchased from Chengdu Pusi Biological Co., including 1,1–diphenyl–2–picrylhydrazyl, 2,2′–azino–bis (3–ethylbenzothiazoline–6–sulfonic acid), choline dihydrogen citrate, choline chloride, betaine, ethylene glycol, glycerol, propylene glycol, lactic acid, malic acid, choline bitartrate, urea, acetic acid, neochlorogenic acid (NA), chlorogenic acid (CA), isochlorogenic acid A (IAA), and isochlorogenic acid C (IAC).

### 2.2 Preparation and screening of TDES

A total of 20 TDESs were prepared in this study by mixing hydrogen bond acceptors (HBAs) and hydrogen bond donors (HBDs) under constant agitation at 70 °C for 1–2 h to achieve homogeneity [18]. A defined amount of water was added as needed. The complete formulation details for the TDESs were presented in Table 1.

**Table 1:** Twenty prepared TDESs.

No.	Abbreviation	HBA:HBD	Molar ratio
DES-1	Chd:Eth:Gly	Choline dihydrogen citrate: ethylene glycol: glycerol	1:2:2
DES-2	Chd:Pro:Gly	Choline dihydrogen citrate: propylene glycol: glycerol	1:2:2
DES-3	Chd:Lac:Gly	Choline dihydrogen citrate: lactic acid: glycerol	1:2:2
DES-4	Chd:Ure:Gly	Choline dihydrogen citrate: urea: glycerol	1:2:2
DES-5	Chd:Ace:Gly	Choline dihydrogen citrate: acetic acid: glycerol	1:2:2
DES-6	Chd:But:Gly	Choline dihydrogen citrate: 1,4-butanediol: glycerol	1:2:2
DES-7	Chd:Lac:Ure	Choline dihydrogen citrate: lactic acid: urea	1:2:2
DES-8	Chc:Ure:Gly	Choline chloride: urea: glycerol	1:2:2
DES-9	Chc:Ure:Ace	Choline chloride: urea: acetic acid	1:2:2
DES-10	Bet:Ure:Gly	Betaine: urea: glycerol	1:2:2
DES-11	Bet:Ure:Lac	Betaine: urea: lactic acid	1:2:2
DES-12	Bet:Ure:Ace	Betaine: urea: acetic acid	1:2:2
DES-13	Bet:Lac:Gly	Betaine: lactic acid: glycerol	1:2:2
DES-14	Chb:Eth:Lac	Choline bitartrate: ethylene glycol: lactic acid	1:2:2
DES-15	Chb:But:Lac	Choline bitartrate:1,4-butanediol: lactic acid	1:2:2
DES-16	Chb:Ace:Lac	Choline bitartrate: acetic acid: lactic acid	1:2:2
DES-17	Chb:Pro:Gly	Choline bitartrate: propylene glycol: glycerol	1:2:2
DES-18	Chb:Lac:Gly	Choline bitartrate: lactic acid: glycerol	1:2:2
DES-19	Chb:Ure:Gly	Choline bitartrate: urea: glycerol	1:2:2
DES-20	Chd:Mal:Gly	Choline dihydrogen citrate: malic acid: glycerol	1:2:2

The extraction was conducted with 3.0 g of *L. japonica* powder dispersed in 60 mL of TDES supplemented with 40% (v/v) water. After initial vortex–mixing for 12 min, the sample was subjected to ultrasound for 30 min under specified conditions (240 W, 40 kHz, 50 °C). Following extraction, the mixture was filtered for further analysis.

### 2.3 Analytical methods

#### 2.3.1 HPLC analysis

**Fig. 1:**
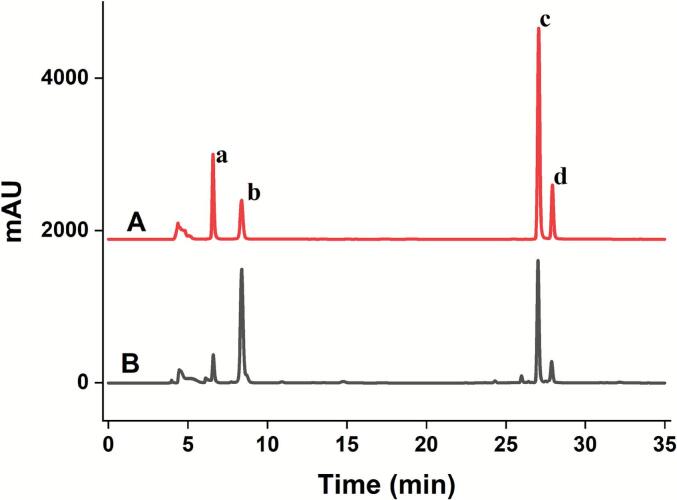
The four standards (A) and *L. japonica* extract (B). The peaks marked with a, b, c, d were NA, CA, IAA and IAC, respectively.

An Agilent 1220 HPLC system coupled with an SB–C18 column (5 μm, 250 × 4.6 mm) was employed for the separation of the four phenolic acid compounds (NA, CA, IAA, IAC). A detailed gradient elution protocol is provided in Table 2. The separation was conducted under the following conditions: column temperature,30 °C; flow rate, 0.6 mL/min; injection volume, 20 μL and detection wavelength, 327 nm. Corresponding chromatograms are shown in Fig. 1.

**Table 2:** HPLC gradient elution.

Time (min)	0.2% phosphoric acid (%)	Acetonitrile (%)
0	82	18
15	82	18
20	71	29
30	71	29
35	55	45

The four phenolic acids yields (%).$=\frac{\text{mean masses of NA,CA,IAA,and IAC (mg)}}{\text{mean masses of L. japonica powder}}\text{[MULSGN]100\%}$

Where the average mass of *L. japonica* powder represented the pre-extraction weight, while the quantities of NA, CA, IAA and IAC were quantified via HPLC analysis.

#### 2.3.2 Chemical profiling of *L. Japonica* extract

The extract of. *japonica* was analyzed by UHPLC–Q–TOF MS for comprehensive chemical characterization. The analysis utilized an Agilent 1290 UHPLC system interfaced with an Agilent 6546 Q–TOF mass spectrometer, fitted with a Waters ACQUITY UPLC HSS T3 column (2.1 × 100 mm, 1.8 μm) for chromatographic separation. The mobile phase composition is detailed in Table S1, with the column maintained at 30 °C, and a 1 μL injection volume.

### 2.4 Optimization of the extraction process

#### 2.4.1 Single–Factor tests and RSM design

To maximize experimental throughput, this work targeted the principal factors affecting bioactive compound extraction. Five critical factors were evaluated to determine the optimal blend for enhancing the phenolic acids’ yield (Table S2). To assess the influence of individual variables on total phenolic acid content, 3.0 g of *L. japonica* powder was combined with the ideal TDES. Each parameter was varied individually while maintaining all others constant [23].

A three–factor, three–level Box–Behnken design (BBD) was constructed to optimize the extraction conditions for maximizing the yield of four phenolic acids. The main variables were presented in Table 3. The resulting model equation is expressed as follows:

$$Y=\beta_{0}+\sum_{i=1}^{i}\left(\beta_{i}x_{i}\right)+\sum_{\mathrm{ij}=1}^{\mathrm{ij}}\left(\beta_{\mathrm{ij}}X_{i}X_{j}\right)+\sum_{1=1}^{i}\left(\beta_{\mathrm{ii}}X^{2}\right)$$

**Table 3:** ANOVA for quadratic model.

Source	Sum of Squares	df	Mean Square	F-value	p-value	significant
Model	2931.84	9	325.76	68.21	< 0.0001	significant
A	2.66	1	2.66	0.5574	0.4796	
B	278.09	1	278.09	58.23	0.0001	
C	510.3	1	510.3	106.86	< 0.0001	
BC	103.31	1	103.31	21.63	0.0023	
A^2^	153.26	1	153.26	32.09	0.0008	
B^2^	286.06	1	286.06	59.9	0.0001	
C^2^	445.54	1	445.54	93.3	< 0.0001	
Residual	582.36	1	582.36	121.94	< 0.0001	
Lack of Fit	403.35	1	403.35	84.46	< 0.0001	
Pure Error	33.43	7	4.78			
Cor Total	5.42	3	1.81	0.2579	0.8528	not significant

Where Y: yield, $\beta_{0}$: constant, $\beta_{\mathrm{ij}}$: coefficients and X: factor.

### 2.5 RSM-ANN-GA

RSM is widely employed for process optimization. Nevertheless, the quadratic-based model is insufficient for modeling the crucial nonlinear interactions inherent in ultrasound-assisted extraction. To address this limitation, a blended RSM–ANN–GA framework was developed [24]. In this framework, systematic training data were first generated using a Box–Behnken design. Subsequently, a multilayer perceptron architecture of an ANN was applied to model the four phenolic acids yield with high–fidelity nonlinear approximation. Finally, a GA was employed to perform global optimization on the response surface predicted by the ANN. By combining both approaches, the model retains the robust experimental basis of RSM while achieving greater accuracy, thus offering a more reliable predictive tool [25].

In the present investigation, rational development and systematic parametric optimization of a BP-ANN model were accomplished via the GA module integrated within MATLAB R2024a Toolbox™ [26], [27]. Model training was based on experimental data derived from RSM, which maps the multivariate input–output relationships governing the extraction system. The adopted training scheme included three pivotal parameters as independent variables, with the objective of predicting the combined yield of four phenolic acids as the dependent variable.

**Fig. 2:**
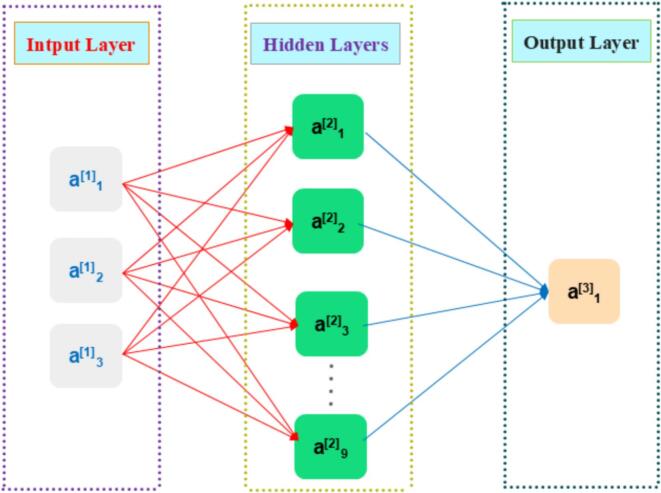
Structure diagram of BP neural network model.

A 3–9–1 artificial neural network (ANN) architecture was developed, comprising three input neurons, nine hidden neurons, and one output neuron, as illustrated in Fig. 2. A methodical sensitivity evaluation refined the architecture, aiming to attain equilibrium between the network’s representational capacity and its out-of-sample performance[28]. Network training was conducted using the Levenberg–Marquardt algorithm, chosen for its computational efficiency in navigating error surfaces during convergence [29]. For rigorous model construction, the complete RSM dataset was strategically split: 70% served as the training set to capture intrinsic patterns, 15% as the validation set for overfitting monitoring, and the remaining 15% as the independent test set. Model selection was guided by the pursuit of minimum MSE and maximum R values, leading to the adoption of the ANN architecture with the best predictive performance. Model parameters were further refined via genetic algorithm optimization, an approach implemented to enhance both the accuracy and stability of predictions.

### 2.6 Comparison with conventional extraction methods

#### 2.6.1 Methanol–Assisted ISUSE

A blend of 3.0 g plant material and 60 mL methanol was subjected to 12 min of vortex mixing, succeeded by 30 min of ultrasonication at 240 W, 40 kHz, and 50 °C. The post–extraction mixture was filtered and then centrifuged at 4000 rpm for 10 min to yield the supernatant. The contents of the four target phenolic acids were subsequently determined by HPLC.

#### 2.6.2 Ultrasound–Assisted methanol extraction (UAE)

A mixture of 3.0 g *L.japonica* powder in 60 mL methanol was sonicated for 30 min at 240 W, 40 kHz, and 50 °C. The resulting mixture was then centrifuged at 4000 rpm for 15 min to collect the supernatant for subsequent analysis.

#### 2.6.3 Maceration extraction (ME)

A 3.0 g sample was placed in a 100 mL flask, combined with 60 mL of methanol, and underwent maceration at 25 °C for 8 h. The resulting mixture was then centrifuged at 4000 rpm for 15 min to collect the supernatant for subsequent analysis.

### 2.7 Mechanistic investigation

#### 2.7.1 FT-IR

The hydrogen bond interactions within the prepared TDES were examined using Fourier–transform infrared spectroscopy. FT-IR spectra (4000–400 cm^−1^) of the selected TDES were obtained.

#### 2.7.2 SEM

To investigate the modifications of different extraction processes on the microstructural characteristics of *Lonicera japonica* powder and further clarify the correlation between the material’s microstructure and the extraction efficiency of its bioactive compounds, scanning electron microscopy (SEM) was employed to examine the surface morphology of the treated *L. japonica* powder.

#### 2.7.3 MD

MD simulations were conducted to investigate the temporal evolution and intermolecular interactions at the atomic level. A comprehensive set of simulation parameters is documented in the Supplementary Material.[20].

### 2.8 Antioxidant activity

#### 2.8.1 DPPH

The DPPH radical scavenging assay was conducted following the protocol established by Qin et al [30]. A series of sample solutions (0.02–0.1 mg/mL) were prepared. Then, 2 mL of each solution was mixed with 2 mL of a methanolic DPPH solution (0.15 mmol/L). The mixture was kept in the dark at room temperature for 30 min, and the absorbance was measured at 517 nm. The scavenging activity was calculated using the following equation:

$$\text{S}\text{cavenging rate}\left(\text{\%}\right)=\left[\frac{\text{Ac-As}}{\text{Ac}}\right]\text{*100\%}$$

Where Ac and As correspond to the absorbance values of the blank control and the sample, respectively.

#### 2.8.2 ABTS

The ABTS radical scavenging assay was conducted following the procedure outlined by Li et al [31]. The ABTS^+^ stock solution was generated by reacting 7 mmol/L ABTS with 2.45 mmol/L potassium persulfate (1:1, v/v) and allowing the mixture to stand in the dark for 16 h. Prior to analysis, this stock was diluted with ethanol to an absorbance of 0.70 ± 0.02 at 734 nm. A mixture was prepared by adding 0.5 mL of sample (0.02–0.1 mg/mL) to 3 mL of the freshly prepared ABTS^+^ reagent. After incubating in the dark for 6 min, the absorbance was recorded at 734 nm. The scavenging activity was calculated according to the following formula:

$$\text{S}\text{cavenging rate}\left(\text{\%}\right)=\left[\frac{\text{A}\text{0}\text{-A}\text{1}}{\text{A}\text{0}}\right]\text{*100\%}$$

Where A_0_ and A_1_ correspond to the absorbance values of the blank control and the sample, respectively.

#### 2.8.3 Ferric ion reducing assay

According to Liu et al [20]. Sample solutions (0.02–0.1 mg/mL) were prepared for analysis. Each sample (3 mL) was reacted with sodium phosphate buffer (2 mL, 0.2 mol/L, pH 6.6) and potassium ferricyanide solution (2 mL, 1%, w/v). The reaction was conducted in a 50 °C water bath for 30 min and then quenched with 2 mL of trichloroacetic acid (10%, w/v). The mixture was then centrifuged at 3000 rpm for 10 min. From the resulting supernatant, 2 mL was transferred and mixed with 0.1 mL of ferric chloride solution (0.1%, w/v) and 2 mL of deionized water. Following a 10–min reaction period, the absorbance was measured at 700 nm.

### 2.9 Statistical analysis

Statistical evaluation of the response surface methodology outcomes was performed through one–way analysis of variance (ANOVA). Experimental data are presented as the mean ± standard deviation. Differences were considered statistically significant at *p < 0.05, **p < 0.01, and ***p < 0.001.

## 3 Results and discussion

### 3.1 Optimization of the TDES formulation

**Fig. 3:**
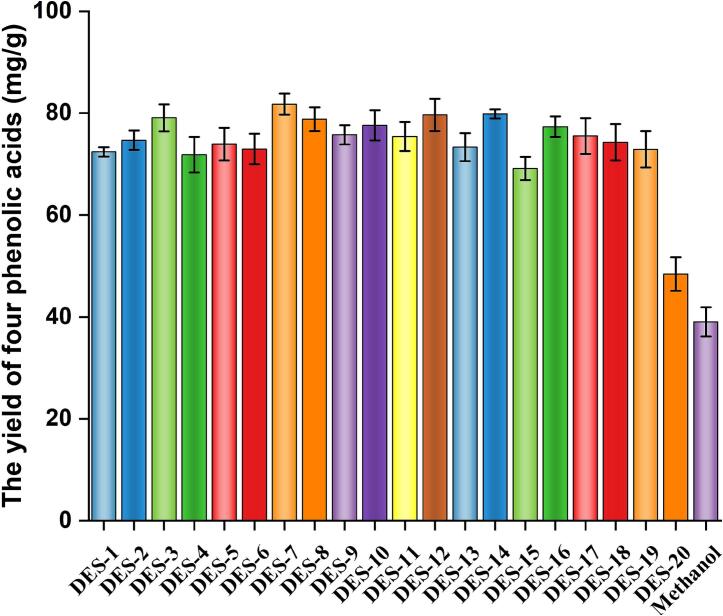
The screen of TDESs.

A total of 20 TDESs were designed for extracting four phenolic acids from *L. japonica*. Systematic screening of these solvents, each containing 30% water, identified clear differences in four phenolic acids extraction efficiency (Fig. 3). The ternary formulation consisting of choline dihydrogen citrate, lactic acid, and urea (Chd:Lac:Ure) performed best. By comparison, methanol extraction yielded 81.76 ± 2.10 mg/g, a result consistent with the “like dissolves like” principle. Given its superior performance, Chd:Lac:Ure was selected as the optimal solvent for all further investigations, aimed at process enhancement and mechanistic elucidation.

### 3.2 Single-factor experimental design

#### 3.2.1 Effect of water content on extraction

The extraction efficiency of TDESs is significantly influenced by their rheological behavior, especially viscosity, which directly affects mass transfer rates. An increase in water content typically reduces TDES viscosity. In this study, adjusting the water content in the selected optimal TDES led to improved extraction yields of the four phenolic acids. Therefore, the effect of varying water content was systematically evaluated to optimize the extraction process.

**Fig. 4:**
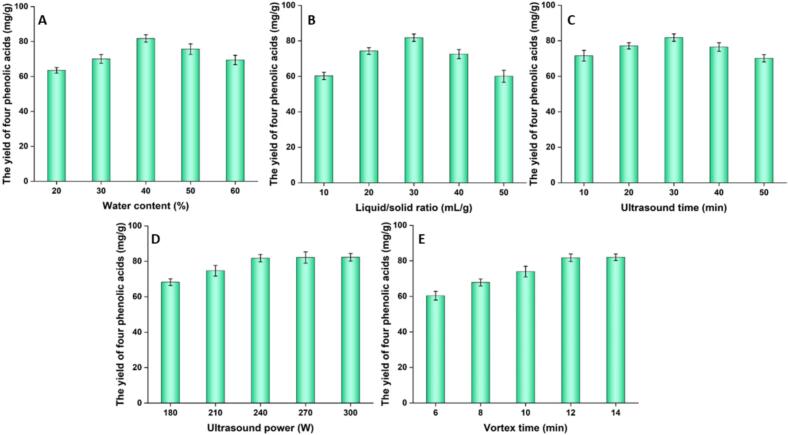
Single factor experiments.

Systematic adjustment of water content revealed a non–linear relationship with the extraction yield of phenolic acids from *L. japonica* (Fig. 4A). The yield increased notably when water content was raised from 20% to 40%, which can be explained by the corresponding decrease in solvent viscosity, thereby enhancing mass transfer and extraction efficiency. However, further increasing water content to 50–60% resulted in a pronounced drop in yield. This decrease can be attributed to a dilution effect, which compromises the hydrogen-bonding network within the DES, thereby diminishing its solvation capacity for the phenolic acids [32].

#### 3.2.2 Effect of other parameters

The liquid/solid ratio directly influences solute diffusion and the extraction driving force. A greater liquid/solid ratio improves mass-transfer efficiency at the expense of higher solvent volumes and potentially elevated impurity levels. As shown in Fig. 4B, the yield rose with increasing liquid/solid ratio, peaking at 30 mL/g. Beyond this point, further increases led to reduced extraction efficiency. This decline can be attributed to two main factors: excessive solvent–herb contact, which promotes the dissolution of competing impurities, and the damping of ultrasound energy propagation in larger solvent volumes, which weakens cell wall disruption and overall extraction performance [22].

The extraction yield was governed by ultrasound time, which directly affects the degree of cell wall disruption via cavitation and subsequent release of target compounds. As shown in Fig. 4C, the yield exhibited a time‑dependent profile, rising progressively until reaching an optimum at 30 min, beyond which a noticeable decrease occurred. This decline may be explained by the prolonged exposure to localized high temperatures generated during bubble collapse, potentially inducing thermal decomposition of the phenolic acids [18].

The efficiency of ultrasound–assisted extraction is closely related to cavitation intensity, which is governed by the input power. As shown in Fig. 4D, the extraction yield increased with rising ultrasound power and reached a maximum at 240 W. Beyond this point, further power increases did not significantly improve the yield, suggesting that the cavitation effect had reached a saturation level where cell–wall disintegration and mass transfer of four phenolic acids were already maximized. This observed plateau can also be ascribed to the attenuation of ultrasonic energy at excessively high power levels, a phenomenon that impairs the effective cavitation efficacy within the solvent medium [12].

Vortex mixing enhances mass transfer during extraction by facilitating efficient dispersion of the botanical matrix in the solvent and promoting the rapid dissolution of bioactive constituents. As shown in Fig. 4E, the yield increased with vortex duration and peaked at 12 min, reflecting effective initial release of phenolic acids. Beyond this point, prolonged mixing did not improve recovery, likely due to the competing dissolution of impurities that may interfere with the solubility equilibrium of the target compounds, leading to a plateau in extraction efficiency [20].

### 3.3 RSM analysis

Optimization of the extraction process was performed using a BBD, focusing on the three critical variables. Design‑Expert software facilitated both the experimental design and data analysis. The experimental variables and corresponding response values are summarized in Table S3. ANOVA of the fitted model confirmed its high statistical significance (p < 0.001, Table 3). The model exhibited a coefficient of determination (*R*^2^) of 0.9887 and an adjusted *R*^2^ of 0.9742, suggesting excellent predictive capability. Furthermore, the lack–of–fit test was non–significant (p > 0.05), indicating that the model adequately describes the experimental data.

The model revealed that the AB, AC, and BC interactions demonstrated synergistic correlations with phenolic acid extraction efficiency, suggesting these parameter combinations may collectively enhance yield. The predictive equation using coded factors is as follows:

$$\text{Y}=\text{82}.\text{52}-0.\text{5768A}+\text{5}.\text{9}0\text{B}-\text{7}.\text{99C}-\text{5}.0\text{8AB}-\text{6}.\text{19AC}+\text{8}.\text{46BC}-\text{1}0.{\text{29A}}^{2}-\text{11}.{\text{76B}}^{2}-\text{9}.{\text{79C}}^{2}$$

**Fig. 5:**
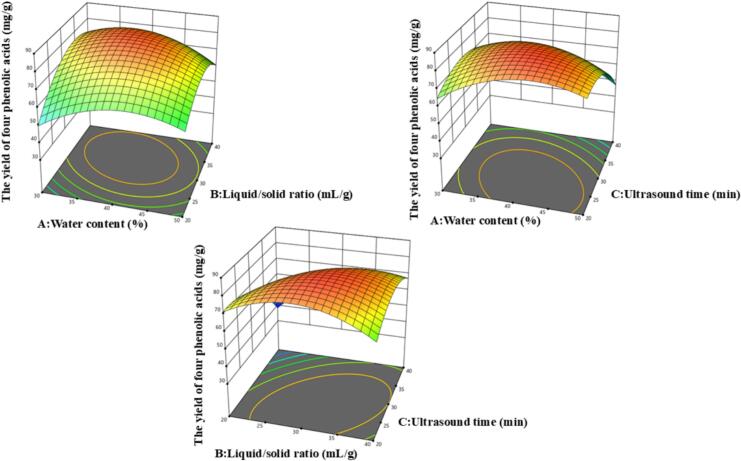
Response surface plots.

The three–dimensional response surfaces (Fig. 5) visually illustrate the combined effects of the investigated variables on four phenolic acids yield. Based on the model predictions, the theoretically optimal conditions were determined as follows: water content, 40.63%; liquid/solid ratio, 30.99 mL/g; and ultrasound time, 26.15 min, corresponding to a predicted maximum yield of 84.33 mg/g. For practical application, these values were rounded to water content 41%, liquid/solid ratio 31 mL/g, and ultrasound time 26 min.

### 3.4 RSM-ANN-GA analysis

**Fig. 6:**
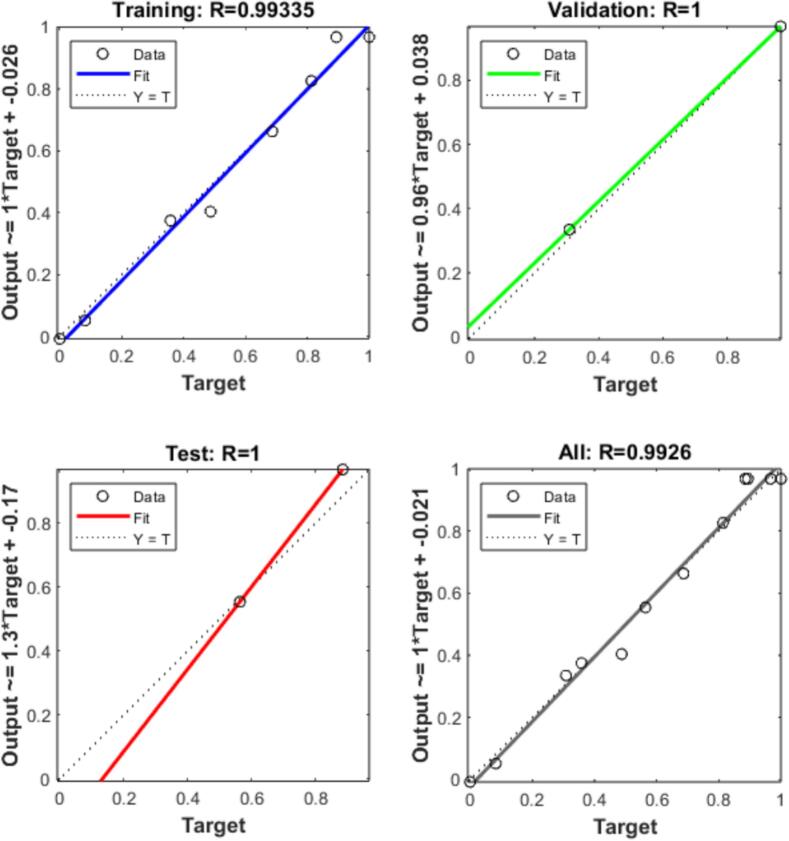
Training, validation, testing and fit of all data to the BP-ANN.

A back–propagation artificial neural network (BP–ANN) was constructed using data generated from response surface methodology. The model architecture incorporated the three independent variables, while the total yield of four phenolic acids was defined as the dependent variable [33]. The network utilized a tansig activation function for the input-hidden connection, followed by a linear function for the hidden-output linkage. Training was performed using the Levenberg–Marquardt backpropagation algorithm (trainlm), with the network configured to run for 1000 epochs, 25 iterations per epoch, and a target mean squared error of 0.0001. Validation performance reached its optimum at epoch 2, exhibiting a mean squared error of 0.00034708 (Fig. S1). The model exhibited strong predictive reliability, as evidenced by correlation coefficients (R) exceeding 0.90 for (Fig. 6).

The optimization objective was defined by the output of the integrated RSM–ANN–GA model. The three independent variables within this setup were defined and bounded as below:

[30; 20; 20] ≤ [X_1_; X_2_; X_3_] ≤ [50; 40; 40].

Based on the integrated RSM–ANN–GA optimization, parameters were derived: water content of 37.10%, liquid/solid ratio of 27.62 mL/g, and ultrasound time of 29.69 min, corresponding to a predicted maximum yield of 90.14 mg/g. To improve experimental practicality and reproducibility, these parameters were adjusted to water content 37%, liquid/solid ratio 28 mL/g, and ultrasound time 30 min for subsequent validation studies.

### 3.5 Validation experiment

As summarized in Table 4, both the RSM and RSM–ANN–GA models demonstrated close agreement with the experimentally determined TF yields. The validation experiment conducted under RSM–optimized conditions produced a yield of 85.62 ± 2.44 mg/g, with a relative standard deviation (RSD) of 2.85%. In comparison, the process optimized by the hybrid RSM–ANN–GA framework achieved a higher yield of 90.66 ± 1.46 mg/g and a lower RSD of 1.62%. These results highlight the superior predictive accuracy and operational stability of the hybrid model, which stems from its enhanced ability to model the complex, nonlinear interactions inherent in the ultrasound–assisted extraction system.

**Table 4:** Validation experiments.

	Water content (%)	Liquid/solid ratio (mL/g)	Ultrasound time (min)	Experimental value	RSD(%)
RSM	41	31	26	85.62 ± 2.44	2.85
RSM-ANN-GA	37	28	30	90.66 ± 1.46	1.62

### 3.6 Comparison with conventional extraction methods

**Fig. 7:**
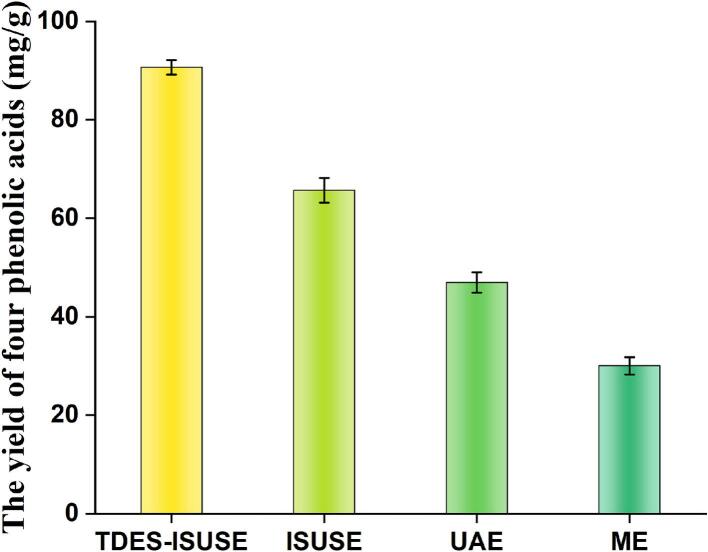
Comparation with four methods.

A comparative analysis was performed to evaluate the efficiency of the developed TDES–ISUSE method for extracting phenolic acids from *L. japonica*. The extraction yield obtained with this method was systematically compared with those from conventional techniques. TDES–ISUSE significantly outperformed ME, requiring markedly shorter extraction time while delivering higher phenolic acid yields(Fig. 7). Furthermore, the yield achieved with TDES–ISUSE exceeded that of ISUSE (65.70 ± 2.52 mg/g) and UAE (46.98 ± 2.07 mg/g). The TDES–ISUSE approach is characterized by rapid operation, superior extraction performance, and eco–friendly attributes, thus holding considerable promise for optimizing the recovery of bioactive constituents from *L. japonica*.

### 3.7 UHPLC-Q-TOF MS analysis

**Fig. 8:**
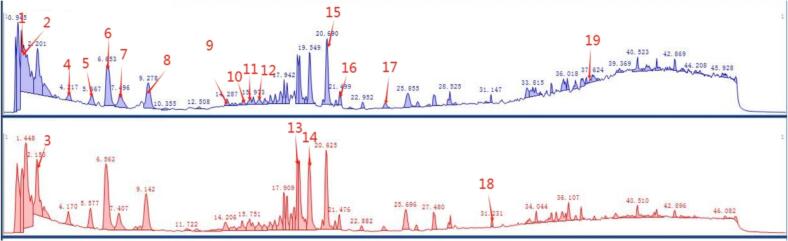
UHPLC–Q-TOF-MS extracted ion chromatograms of the compounds from *L. japonica*. positive (A) and negative(B) ion modes.

UHPLC–Q–TOF–MS was applied to characterize chemical constituents of *L. japonica* extract, capitalizing on its high resolution, mass accuracy, and sensitivity for natural product analysis [34]. This work represents the first comprehensive chemical profiling of TDES extract using this platform, aiming to delineate its bioactive composition. Total ion chromatograms obtained in positive and negative ionization modes are shown in Fig. 8. Integration of MS/MS fragmentation patterns with the PCDL database and published literature led to the annotation of 19 compounds, categorized as seven phenolic acids, three flavonoids, one iridoid, one terpenoid, five saponins, and two fatty acids (Table 5). Among these, peaks 1, 2, 5, and 6 were confirmed as NA, CA, IAA and IAC, respectively. The detailed chemical profile established here offers a reliable foundation for further pharmacological exploration and product development of *L. japonica*.

**Table 5:** The result of UHPLC-Q-TOF MS.

Peak	RT(min)	Model	Compound	Molecular formula	Adduct (*m*/*z*)	Major fragment ion	References
1	1.207	+	Neochlorogenic acid	C16H18O9	354.0951	163.0398,145.0285,135.0442,117.0335	[35]
2	1.406	+	Chlorogenic acid	C16H18O9	354.0951	163.0398,145.0285,117.0335	[35], [36]
3	2.201	−	Kingiside	C17H24O11	404.1319	371.0982,223.0612,179.0561,121.0244	[37]
4	4.217	+	Isoquercitrin	C21H20O12	464.0955	303.0503,229.0500,165.0184,145.0496	[38]
5	5.667	+	Isochlorogenic acid A	C25H24O12	516.1268	499.1233,337.0917,319.0813,163.0392	[35], [39]
6	6.653	+	Isochlorogenic acid C	C25H24O12	516.1268	499.1238,337.0919,319.0814,163.0393	[35]
7	7.496	+	Isorhamnetin-3-O-beta-D-Glucoside	C22H22O12	478.1111	317.0660,229.0492,153.0182,145.0497	[40]
8	9.278	+	Isochlorogenic acid B	C25H24O12	516.1268	499.1237,337.0919,319.0814,163.0394	[35], [41]
9	14.287	+	4,5-Di-O-caffeoylquinicacidmethyl ester	C26H26O12	530.1425	319.0819,177.0545,163.0390,145.0278	[41]
10	15.318	+	1,4-Dicaffeoylquinic acid	C25H24O12	516.1265	499.1225,355.1020,319.0824,163.0390,135.0437	[42]
11	15.786	+	helichrysoside	C30H26O14	610.1323	309.0968,291.0863,165.0546,147.0442	[43]
12	16.348	+	Borneol 7-O-[β-D-apiofuranosyl-(1 → 6)]-β-D-glucopyranoside	C21H36O10	448.2308	296.0811,193.0506,137.1325,115.0389,109.0280	[44]
13	17.942	−	Macranthoidin B	C65H106O32	1398.6667	1073.5534	[45]
14	19.549	−	Macranthoidin A	C59H96O27	1236.6139	1074.0552,911.5013,749.4475,603.3901	[46]
15	20.690	+	Nigelloside C	C53H86O22	1074.5611	943.5235,619.4192,455.3518,309.1184	[47]
16	21.499	+	Akebia saponin D	C47H76O18	928.5032	767.4560,635.4146,455.3515,295.1021,207.1744	[47]
17	24.404	+	10-Octadecenoic acid,9,12,13-trihydroxy-, (9R,10E,12R,13S)	C18H34O5	330.2406	295.2273,277.2163,195.1157,155.1068	[48]
18	31.147	−	Dipsacoside B	C53H86O22	1074.5611	911.4970,749.4464,603.3898,471.3473	[47]
19	37.472	+	9(Z),11(E)-Octadecadienoic acid	C18H30O3	294.2195	277.2163,259.2054,179.1431,135.1168	[49]

### 3.8 Method validation

HPLC analysis confirmed the presence of four principal phenolic acids in *L. japonica* extracts: NA, CA, IAA and IAC. To precisely assess the quality of the extracts, these compounds were quantified under the optimized extraction protocol. The reliability of the quantitative method was verified by constructing calibration curves using reference standards, which exhibited good linearity across defined concentration ranges, as reflected by high correlation coefficients (R^2^).

The standard curves of NA, CA, IAA and IAC were Y_NA_ = −503.63x + 2917.1(R^2^ = 0.9992), Y_CA_ = −962.38x + 5424.1 (R^2^ = 0.9993), Y_IAA_ = −799.68x + 4596.6 (R^2^ = 0.9991), Y_IAC_ = −756.33x + 4400.9 (R^2^ = 0.9999), respectively. The four target phenolic acids displayed a wide linear dynamic range (6.25–100.00 μg/mL), with correlation coefficients (R^2^) greater than 0.999 for all calibration curves, indicating excellent linearity(Table 6). The limits of detection (LOD) and limits of quantification (LOQ) for these compounds were in the ranges of 0.39–0.48 μg/mL and 1.18–1.40 μg/mL, respectively, confirming the high sensitivity and suitability of the established HPLC method for quantitative analysis.

**Table 6:** Method validation.

Analyte	LOD (μg/ mL)	LOQ (μg/mL)	Intraday precision	Interday precision	Stability	Repeatability	Recovery
NA	0.47	1.31	0.84	1.46	1. 68	1.82	99.63
CA	0.39	1.18	0.79	1.53	1.74	1.26	99.52
IAA	0.42	1.22	0.62	1.82	1.93	1.64	98.87
IAC	0.48	1.40	0.70	1.91	1.57	1. 88	99.95

Method precision was evaluated by analyzing the same sample six times within a single day (intraday) and once daily over six consecutive days (interday). The RSD for both intra-day and inter-day measurements was less than 2%, confirming the high reproducibility of the analytical procedure. Sample stability was further investigated over a 12–h period at 25 °C, with testing intervals at 0, 3, 6, 9, and 12 h. The RSD values remained below 2% throughout this duration, demonstrating excellent short–term stability of the prepared extracts under the tested conditions.

Method repeatability was confirmed by analyzing six replicate samples, with RSD values between 1.26% and 1.88%, indicating excellent reproducibility. Recovery tests were performed by spiking a known amount of the phenolic acid standards into 3.0 g of raw plant material. The resulting recovery rates are summarized in Table 6, further verifying the accuracy of the analytical method.

## 9 Extraction mechanism

### 3.9.1 FT-IR

TDESs consist of a HBA paired with two HBDs. In this work, the molecular interactions and structural evolution during TDES formation were analyzed by FT–IR spectroscopy, comparing the spectra of individual components (choline dihydrogen citrate, lactic acid, and urea) with that of the synthesized ternary mixture.

**Fig. 9:**
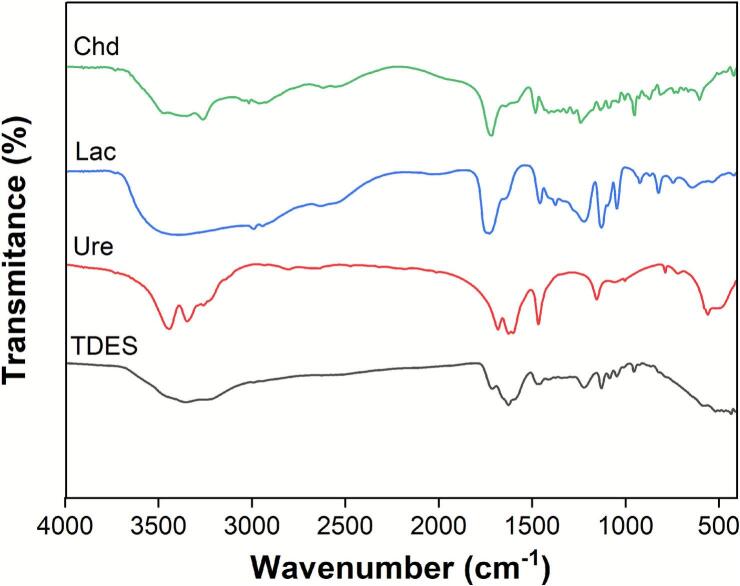
FT-IR spectra of choline dihydrogen citrate, lactic acid, urea and TDES.

Key functional group regions were identified in the spectra (Fig. 9): a broad band at 3500–3200 cm^−1^ (–OH stretching), signals between 3000–2500 cm^−1^ (C–H vibrations), and a distinct region around 1112–1000 cm^−1^ (C–O stretching). Notably, the C–N stretching vibration of choline dihydrogen citrate, observed in the TDES spectrum, shifted to 1211 cm^−1^ [12], [20]. Moreover, the –OH absorption band in the TDES was markedly broader and exhibited a blue shift relative to the individual components. These characteristic spectral shifts validate the formation of an extensive hydrogen–bonding network, confirming the successful preparation of the TDES.

### 3.9.2 SEM

**Fig. 10:**
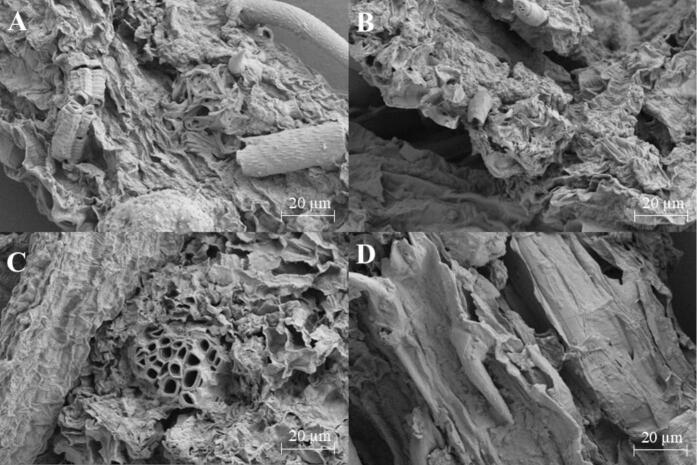
SEM results of *L.japonica* powders (A: TDES-ISUSE, B: ISUSE, C: UAE and D: ME).

This study adopted a combined vortex–ultrasonication strategy to enhance extraction efficiency. Vortex mixing generates pronounced turbulence at the solid–liquid interface, accelerating solvent penetration and the initial release of target compounds from the plant matrix [50]. Cavitation, driven by alternating compression–rarefaction cycles in the liquid phase during ultrasonication, produces mechanical shear forces sufficient to compromise cellular integrity. The integration of these two mechanisms yields a synergistic effect: vortex–induced convective mass transfer facilitates solvent access, while ultrasound–mediated cavitation intensifies cell–wall disintegration, collectively leading to more complete liberation of intracellular constituents. The enhanced structural disruption achieved with this combined approach is visually confirmed by the microscopic images presented in Fig. 10A.

Plant cell walls are predominantly composed of cellulose, a crystalline polysaccharide whose efficient dissolution is critical for effective extraction. TDES, formed by HBA and HBDs, exhibit a superior capacity to disrupt cellulose compared to conventional solvents. This efficacy arises from their ability to engage in selective hydrogen–bonding interactions with cellulose chains, cooperatively breaking the intrinsic hydrogen–bond network and reducing crystallinity, thereby promoting solubilization. As visually demonstrated in the SEM images (Fig. 10AA–D), plant material treated with the TDES–ISUSE method showed substantially more pronounced cell wall breakdown than samples processed by conventional techniques. This enhanced structural disruption directly correlates with the improved extraction efficiency of target bioactive compounds, confirming the mechanistic role of the ternary DES in facilitating biomass deconstruction and compound release.

### 3.9.3 MD analysis

To uncover the mechanistic basis for the enhanced extraction of phenolic acids from *L. japonica* using the TDES–ISUSE method, MD simulations were performed. The simulations compared the most effective TDES (Chd:Lac:Ure) with methanol, focusing on their molecular–level interactions with CA, a major phenolic acid in this herb. The analysis centered on key intermolecular forces—such as hydrogen bonding, van der Waals contacts, and electrostatic interactions—that directly influence solute solubility and partitioning. The simulation results demonstrated that the TDES formed a more extensive and stable interaction network with EC than methanol, providing a molecular–scale explanation for the higher extraction efficiency observed experimentally.

**Fig. 11:**
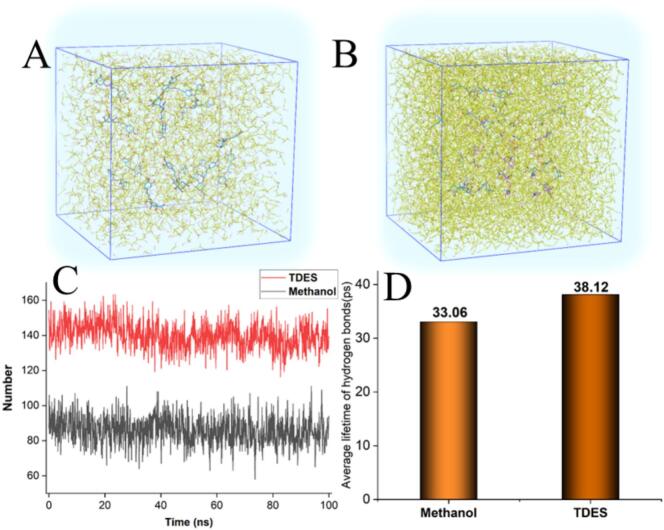
Snapshots of CA dissolved in Methanol (A) and TDES (B); the average lifetime of hydrogen bonds (C) and average lifetime of hydrogen bonds (D).

Molecular dynamics simulations provided direct visualization of the distinct solvation behaviors of chlorogenic acid (CA) in the ternary DES versus methanol. After 100 ns, CA exhibited a more uniform spatial distribution within the TDES system, in contrast to its aggregation in methanol (Fig. 11A, B), which correlates with its higher experimental solubility. Quantitative analysis further revealed that CA formed a greater number of hydrogen bonds with the TDES, and these interactions were significantly more stable, as evidenced by both the consistently higher hydrogen–bond count (Fig. 11C) and prolonged bond lifetimes (Fig. 11D). Collectively, these simulation results confirm at the molecular level that the TDES creates a more favorable solvation environment, thereby providing a mechanistic foundation for its superior extraction performance.

#### Antioxidant activity

**Fig. 12:**
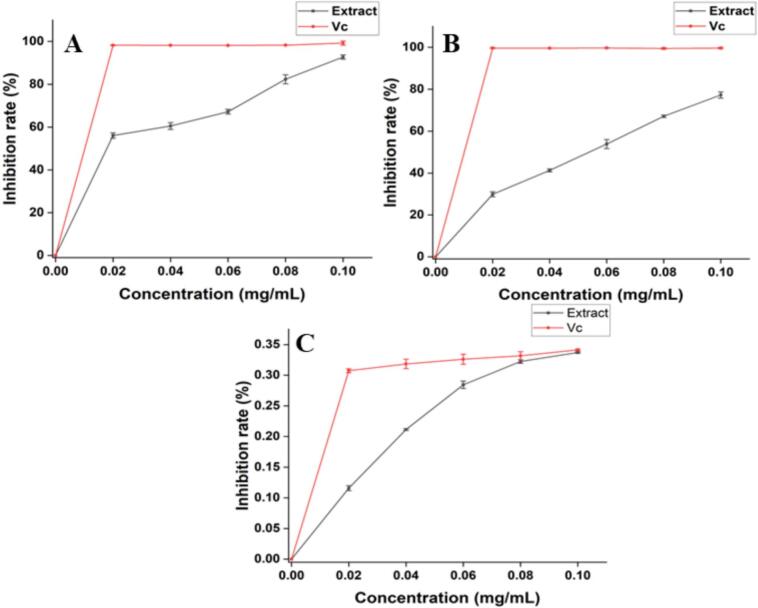
Antioxidant activities (a) DPPH; (b) ABTS; (c) reducing ability.

The antioxidant potential of *L. japonica* extracts was systematically assessed using three complementary in vitro assays: DPPH^+^ and ABTS^+^ radical scavenging, and ferric ion reducing power. As shown in Fig. 12A and B, the extract displayed concentration–dependent scavenging activity against both radicals within the range of 0.02–0.1 mg/mL. At the highest concentration (0.1 mg/mL), scavenging rates reached 92.73 ± 1.01% for DPPH^+^ and 77.28 ± 1.48% for ABTS^+^, demonstrating strong radical–neutralizing capacity. Ascorbic acid, used as a positive control, exhibited scavenging rates above 90% in both assays.

The reducing power of the extract, reflecting its ability to reduce Fe^3+^ to Fe^2+^, was also confirmed (Fig. 12C). At 0.1 mg/mL, the absorbance reached 0.337 ± 0.001, indicating notable electron–donating activity. Collectively, these results highlight the potent antioxidant properties of *L. japonica* extracts, supporting their potential application as natural antioxidants in functional foods or phytopharmaceuticals.

## 4 Conclusion

A TDES-ISUSE approach, based on a newly formulated natural ternary deep eutectic solvent, was designed and applied to achieve efficient phenolic acid extraction from *L. japonica*. After systematic screening, the TDES composed of choline dihydrogen citrate, lactic acid, and urea (Chd:Lac:Ure) was selected as the optimal solvent. Process optimization using an integrated RSM–ANN–GA framework yielded the following conditions: water content 37%, liquid/solid ratio 28 mL/g, ultrasonication time 30 min, vortex time 12 min, and ultrasound power 240 W, under which the total phenolic acid yield reached 90.66 ± 1.46 mg/g—significantly higher than conventional extraction techniques. UHPLC-Q-TOF-MS analysis enabled the unambiguous identification of 19 phytochemicals, which were classified into six categories including seven phenolic acids, three flavonoids, one iridoid, one terpenoid, five saponins and two fatty acids. In addition, a fully validated HPLC method was established for the simultaneous quantitative determination of the four principal phenolic acids in the sample. FT–IR confirmed TDES formation, SEM revealed heightened cell–wall disruption, and MD simulations illustrated reinforced solvation of chlorogenic acid in the TDES system. In vitro antioxidant assays indicated strong radical–scavenging activity, supporting the potential use of the extract in functional foods or pharmaceuticals.

Collectively, the TDES-ISUSE extraction method stands as an environmentally benign and highly efficient green extraction approach for the isolation of bioactive phytochemicals from medicinal plant matrices. This integrated technological strategy synergistically capitalizes on the intrinsic eco-friendly merits of ternary deep eutectic solvents and the enhanced mass transfer efficiency of the ISUSE technique, which enables the achievement of high yields of target bioactive components from medicinal plant raw materials and thus furnishes a practical and promising alternative for the sustainable extraction of bioactive constituents in natural product and phytopharmaceutical research.

## CRediT authorship contribution statement

**Cheng Liu:** Writing – original draft, Investigation, Funding acquisition. **Qian Zhao:** Formal analysis. **Xin Ma:** Investigation. **Shuxin He:** Investigation. **Xiaochuan Zou:** Investigation. **Fangyuan Gong:** Software. **Ying Liu:** Software. **Jie Lei:** Writing – review & editing, Methodology. **Zhengwei Xiong:** Project administration. **Jiayun Liu:** Project administration.

## Declaration of competing interest

The authors declare that they have no known competing financial interests or personal relationships that could have appeared to influence the work reported in this paper.
