# Supplementary material for: Novel green solvent-in situ ultrasound synergistic extraction of phenolic acids from Lonicera japonica thunb
Source: Ultrason Sonochem. 2026 Feb 13;127:107779. doi: 10.1016/j.ultsonch.2026.107779 (PMC12930038; doi:10.1016/j.ultsonch.2026.107779)
Supplement: Supplementary Data 1 [file mmc1.docx]

Novel Green Solvent-In Situ Ultrasound Synergistic Extraction of Four Phenolic Acids from *Lonicera japonica* Thunb

Cheng Liu^a,b^, Qian Zhao^b^, Xin Ma^b^, Shuxin He^b^, Xiaochuan Zou^b^, Fangyuan Gong^b^, Ying Liu^b^, Jie Lei^a,b*^, Zhengwei Xiong ^a,b,c*^, Jiayun Liu^c*^

a Chongqing Field Scientific Observation and Research Station for Authentic Traditional Chinese Medicine in the Three Gorges Reservoir Area, Chongqing University of Education, Chongqing 400067, China

b College of Biological and Chemical Engineering, Chongqing University of Education, Chongqing 400067, China;

c Department of Clinical Laboratory, Xijing Hospital, Fourth Military Medical University, Xi’an 710032, China

* Corresponding author:

**2.** **Materials and methods**

2.7.3 MD analysis

Molecular dynamics simulations were conducted using GROMACS, a robust molecular dynamics package designed for simulating large molecular systems. The simulations were performed to investigate the dynamics and interactions of molecules in two different solvents over a 100-nanosecond (ns) period. The OPLS-AA force field was employed to accurately model the intermolecular interactions within the system. For the solvents, we used the TIP3P water model for Methanol and a custom solvent model for TDES, with optimized parameters to ensure realistic simulation conditions.

The simulations were carried out at a temperature of 300 K and a pressure of 1 atm. Each production run lasted for 100 ns, using a time step of 2 fs. The workflow began with system preparation, where the molecule and solvent structures were combined and energy-minimized to eliminate steric clashes and optimize initial positions. This was followed by a 100 ps NVT (constant number of particles, volume, and temperature) equilibration to stabilize the temperature, and a 100 ps NPT (constant number of particles, pressure, and temperature) equilibration to stabilize the pressure and volume. The production run was then executed in the NPT ensemble, with data collected at a frequency of 1 ps throughout the simulation.

The results of these simulations are presented in the attached PPT document, where the final (100 ns) snapshots of the systems in Methanol and TDES are shared. Significant changes in molecular configurations and solvent-molecule interactions were observed over the 100 ns simulation period for both solvents. Detailed analysis of the trajectories revealed important insights into the dynamic behavior of the systems, with visualizations including molecular snapshots, radial distribution functions, and mean squared displacement plots illustrating the evolution of the systems over time.

Table S1 UHPLC gradient elution

| Time (min) | acetonitrile (%) | 0.1% aqueous formic acid (%) |
| --- | --- | --- |
| 0 | 18 | 82 |
| 10 | 18 | 82 |
| 16 | 29 | 71 |
| 24 | 29 | 71 |
| 30 | 45 | 55 |
| 40 | 90 | 10 |
| 45 | 90 | 10 |
| 45.1 | 18 | 82 |
| 50 | 18 | 82 |

Table S2 Experimental ranges and levels in single-factor experiment.

| Factors | Units | Level | | | | |
| --- | --- | --- | --- | --- | --- | --- |
| A: water content | % | 20 | 30 | 40 | 50 | 60 |
| B: liquid/solid ratio | mL/g | 10 | 20 | 30 | 40 | 50 |
| C: ultrasound time | min | 10 | 20 | 30 | 40 | 50 |
| D: ultrasound power | W | 180 | 210 | 240 | 270 | 300 |
| E: vortex time | min | 6 | 8 | 10 | 12 | 14 |

Table S3 Box-Behnken experimental results.

| No. | A (%) | B (mL/g) | C(min) | Y(mg/g) |
| --- | --- | --- | --- | --- |
| 1 | 40 | 40 | 40 | 67.98 |
| 2 | 40 | 20 | 40 | 39.46 |
| 3 | 30 | 30 | 20 | 65.19 |
| 4 | 40 | 30 | 30 | 83.98 |
| 5 | 30 | 40 | 30 | 72.48 |
| 6 | 30 | 20 | 30 | 50.34 |
| 7 | 40 | 30 | 30 | 86.02 |
| 8 | 50 | 30 | 20 | 77.14 |
| 9 | 40 | 30 | 30 | 80.08 |
| 10 | 30 | 30 | 40 | 60.13 |
| 11 | 40 | 30 | 30 | 79.76 |
| 12 | 50 | 40 | 30 | 60.44 |
| 13 | 40 | 40 | 20 | 65.57 |
| 14 | 40 | 30 | 30 | 82.77 |
| 15 | 50 | 20 | 30 | 58.63 |
| 16 | 40 | 20 | 20 | 70.88 |
| 17 | 50 | 30 | 40 | 47.32 |


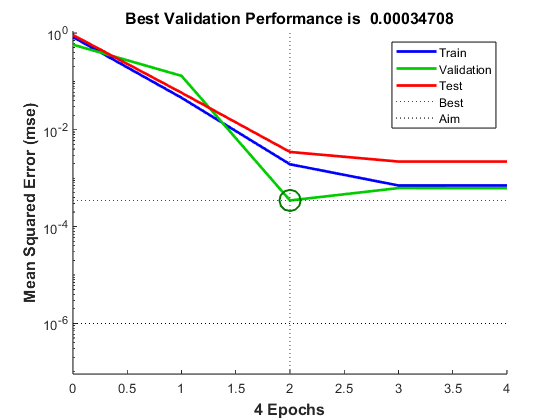


Fig.S1 BP-ANN training performance
